# E-Handover in Surgery Improves Clinical Efficiency and Adherence to COVID-19 Infection Control Measures

**DOI:** 10.7759/cureus.13967

**Published:** 2021-03-18

**Authors:** Neville Jacob, Osman Chaudhary, Nourelhuda M Darwish, Vardhini Vijay, Helen Pardoe

**Affiliations:** 1 Surgery, Princess Alexandra Hospital, Harlow, GBR

**Keywords:** covid-19, electronic handover, e-handover, information governance, efficiency, infection control

## Abstract

Introduction

The ongoing coronavirus disease 2019 (COVID-19) pandemic has impacted all aspects of clinical practice. A district general hospital's surgical department identified that ward rounds based on a paper-based handover system did not adhere to good COVID-19 pandemic infection control measures, including social distancing, reduction of footfall, and reducing contact events during documentation. Surgical E-Handover was introduced as a quality improvement project focussing on increasing efficiency and improving patient safety and compliance with COVID-19 social distancing measures. Other objectives were to reduce the risk of information governance breaches. During the COVID pandemic, there was a significant investment in digital technology, which supported rapid advancement in the use of electronic healthcare solutions to deliver new ways of working. We used the opportunity of the emergency situation to disrupt existing work patterns and introduce surgical E-Handover.

Methods

A quality improvement team of stakeholders was assembled, and a project to introduce E-Handover was carried out using the trust quality improvement methodology aligned to the Institute of Healthcare Improvement (IHI). Questionnaires were sent out pre- and post-implementation to evaluate the impact of using E-Handover during ward rounds.

Results

The efficiency of ward rounds was improved and improving compliance with COVID 19 social distancing measures was highly successful. These outcomes were achieved by reducing footfall during ward rounds, as key clinical information was available at the bedside (p<0.001). Doctors spent less time in crowded clinical multi-disciplinary team (MDT) rooms, and the integrated paper healthcare records were not accessed by multiple staff members simultaneously. The implementation of the E-Handover improved the safety and efficiency of the surgical department, particularly with reference to potential information governance breaches (p<0.001).

Conclusion

Surgical E-Handover, as compared to a printed patient list, significantly improved clinical efficiency and adherence to COVID-19 social distancing measures. E-Handover should be routinely used in surgical ward rounds.

## Introduction

Problem

An increasingly ageing population has placed greater pressures on all aspects of the health service, with increased patient flow resulting in a greater administrative burden on clinicians and allied healthcare professionals. The European Working Time Directive (EWTD) has directly impacted patient continuity of care and the consequence is ever more reliance on frequent and robust handovers [1].

The British Medical Association (BMA) and the World Health Organisation (WHO) highlight poor handovers to be a leading contributing factor to patient harm [1-2]. The General Medical Council (GMC) has highlighted this requirement [3], and The Royal College of Surgeons of England (RCS England) has encouraged surgeons to follow the GMC guidelines to ensure patient safety is not compromised [4].

As healthcare professionals, there is also a duty to patients to maintain health information privacy as per the General Data Protection Regulation and the Data Protection Act 2018.

Infection control measures in a pandemic include recommendations to introduce social distancing rules, reduce footfall in a health care environment and minimise all contact with any surface. The use of a printed patient list in surgical ward rounds was reviewed. On discussion, the surgical handover was deemed inefficient, ward rounds were continually interrupted, information governance laws were sometimes breached and doctors were frequently crowded in multi-disciplinary team (MDT) rooms. An updated model for surgical handover and ward rounds was proposed.

Background

The ongoing coronavirus disease 2019 (COVID-19) pandemic has impacted all aspects of clinical practice. More than 50 million cases of COVID-19 have been reported in more than 200 countries and territories, resulting in over 1.2 million deaths [5]. The virus is most often spread via small droplets from infected individuals coughing, sneezing and talking [6]. Therefore, preventative measures include hand washing, covering one’s mouth when coughing, maintaining distance from other people, wearing a face mask in public settings and monitoring and self-isolating for people who suspect they are infected [7]. After contrasting evidence of risk of fomite transmission of COVID-19, The Lancet stated that while the chance of spread is small, touching a surface ‘within 1-2 hours’ of an infected person coughing or sneezing on the surface can lead to transmission [8]. Following these policies poses challenges for doctors as they perform handovers and take part in regular ward activity. Social distancing may act as a barrier for information transfer from one team to another, thus increasing the risk of misinformation leading to increased patient harm.

Princess Alexandra Hospitals NHS Trust (PAHT) is a 419-bed district general hospital in Harlow, Essex, United Kingdom, with 74 surgical inpatient beds. The trust has approximately 101,000 attendances in the emergency department per year. There were 17,758 surgical admissions between November 2017 and October 2018. Emergency admissions accounted for 6,091 (34.3%), 9,704 (54.6%) were day cases, and the remaining 1,963 (11.1%) were elective cases.

As is routine in many hospitals, PAHT conducts surgical handover twice a day, at 08:00 and 20:00. Doctors perform handover and clinical work in a shared clinical administration area called the multi-disciplinary team (MDT) room. It is the responsibility of the on-call surgical team to compile a patient list with details of all patients referred to surgery into a standardised template. On a daily basis, juniors belonging to individual teams compile a list of patients belonging to the individual consultants responsible for their care.

Prior to our intervention, all the patient lists were created and stored on an internal X: drive using a Microsoft Word^TM^ (Microsoft Corporation, Redmond, WA) template. This document would be printed and distributed to all team members prior to handover, often leading to >12 copies of patient lists printed per day. Sometimes, the paper lists would not be disposed of in the confidential waste immediately, may be left in a public place or occasionally accidentally taken offsite, leading to information governance breaches.

The hospital had already invested in NerveCentre^TM^ software (Nervecentre Software Ltd, Wokingham, UK), which was widely used by all clinical staff in order to record patient observations. NerveCentre^TM^ is available to all appropriately trained clinical staff who have authorised accounts, and it has passed all Trust Data Protection Impact Assessment requirements. Within the software, an electronic handover (E-Handover) template existed but was not routinely used in the hospital.

The aim of the study was to prospectively audit the introduction of surgical E-Handover in ward rounds with a particular focus on increasing the efficiency of surgical handover and ward rounds and improving information governance. Secondary outcome measures were improving adherence to government advice regarding COVID-19 pandemic social distancing measures.

## Materials and methods

NerveCentre^TM^ is able to provide sections for COVID-19 Status, Reason for Review, Working Diagnosis, Issues, Management Plan and Relevant Past Medical History for all inpatients. Clinical staff are able to view patient information individually or by location, speciality or responsible consultant. An E-Handover surgical template document was designed using the NerveCentre^TM^ resources already established. There is also the facility to set up bespoke lists.

A baseline questionnaire was sent out on May 6, 2020, to all doctors in the surgical department to evaluate surgical handover and ward rounds using printed patient lists, patient ward round behaviour and adherence to COVID-19 pandemic infection control measures (see Appendix A).

After analysis of the results and consultant surgeon engagement, a team of five doctors (Foundation Doctor, Senior House Officer, Registrar, two Consultants) introduced NerveCentre^TM^ and iPads to use for surgical E-Handover. These replaced the existing Microsoft Word^TM^ template with the NerveCentre^TM ^template (see Appendices B and C). Training documents and written flowcharts were used to help educate the rest of the surgical team on using NerveCentre^TM^ software. Apart from NerveCentre^TM^, other key clinical systems were also available on the iPad, including limited clinical noting, clinical laboratory reports, radiology images and reports and E prescribing.

Our primary endpoint was to make a statistically significant improvement in the efficiency of surgical ward rounds and an improvement in information governance at our hospital by using surgical E-Handover (p<0.05). 

Our secondary endpoint was for 100% of doctors to feel as if they were able to follow COVID-19 pandemic social distancing measures in the clinical workspace.

We measured this by looking at factors influencing the efficiency of ward rounds and how well the doctors were able to maintain social distancing, particularly during ward rounds. This included whether doctors returned to the MDT room to check patient information, whether they were required to be in crowded MDT rooms and whether they needed to use multiple computers.

E-Handover was introduced in two phases. Phase 1, which started on May 13, 2020, was a hybrid system where the acute admissions were managed using E-Handover and the inpatient handover was using printed paper lists. Phase 2, which started a week later on May 20, 2020, transferred all surgical handover to the E-Handover format.

We followed the Standards of Quality Improvement Reporting Excellence (SQUIRE) guidelines to increase the completeness and transparency of reporting our quality improvement project [9].

## Results

Thirty-seven responses were received to the baseline questionnaire (92.5% response rate) (see Appendix D). The repeat questionnaire, which was sent two months after the beginning of the project, received 23 responses (57.5% response rate).

**Figure 1:**
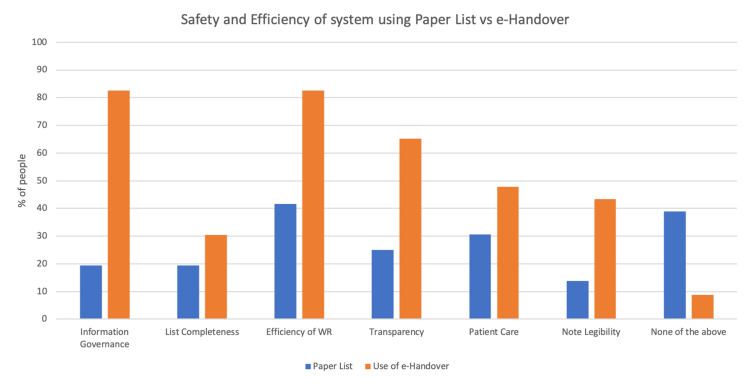
Safety and efficiency of the surgical department using Paper Lists vs E-Handover

After the introduction of Surgical E-Handover and the use of iPads during ward rounds, 41% more doctors reported an improvement in the efficiency of ward rounds (p=0.002) (Figure 1).

**Figure 2:**
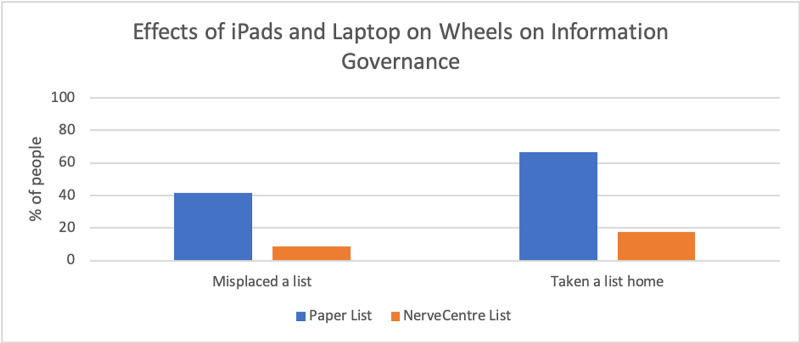
Effects of iPads and laptop on wheels on information governance

Sixty-three per cent (63%) of doctors noticed an improvement in information governance (p<0.001) (Figure 1) while using the E-Handover, with a 33% decrease in doctors misplacing a printed paper patient list (p<0.006) and a 49% decrease in doctors accidentally taking a printed list containing patient information home (p<0.001) (Figure 2).

Our secondary endpoint was for 100% of doctors to feel as if they were able to maintain social distancing when appropriate in a hospital setting.

**Figure 3:**
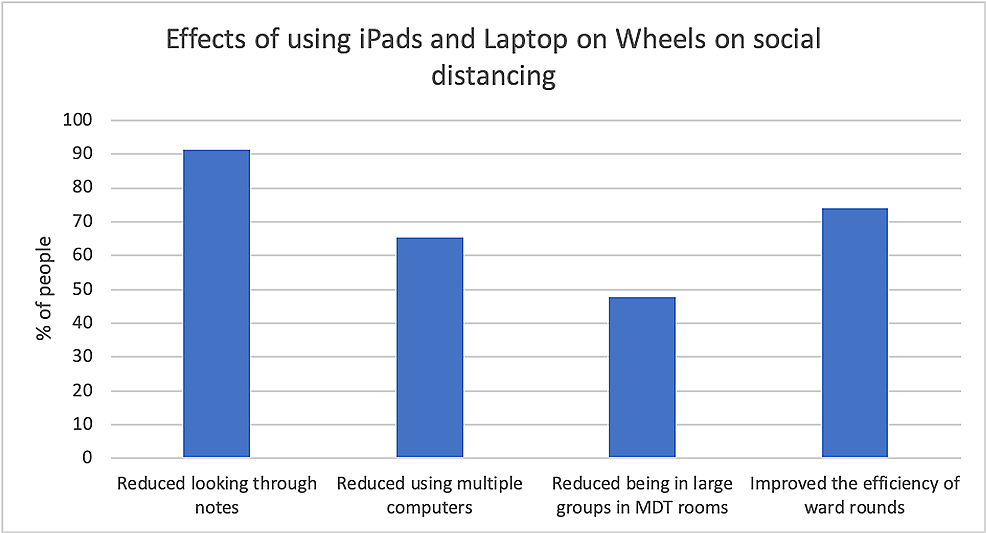
Effects of using E-Handover on social distancing

When able to use the iPads and laptops during ward rounds, more than 90% of doctors reduced the frequency with which they needed to spend time looking through the paper integrated health care records, 65% noted that they were not using multiple computers and, most importantly, 48% reported that the implementation had reduced doctors’ need of being in large groups in busy MDT rooms (Figure 3).

Regarding patient care, 21% and 17% more doctors felt improvement with using the iPads and E-Handover respectively (Figure 1).

Prior to the implementation of surgical E-Handover using mobile devices, over 75% of doctors had returned to the MDT room to review clinical laboratory or radiology results or medications. 97% of doctors said that they would find it beneficial to have this information at the patient bedside. After introducing the E-Handover to the department, these numbers decreased significantly, as this information was now readily available at the patient bedside (p<0.001); 73% decrease in checking blood tests, 69% decrease in checking scans, 67% decrease in checking observations and a 76% decrease in doctors checking patient medications. There was also a 34% decrease in doctors needing to return to the MDT room to check past medical history (PMHx). When this information was made available, 75% of doctors reported a change in a patient’s management plans. By using the E-Handover, 65% of doctors reported that they had up-to-date information at the patient bedside (Table 1).

**Table 1:** Comparison between Paper Lists vs E-Handover

		Paper List	NerveCentre List	P-value
Needing to return to the MDT room to check patient information	Bloods	77.78%	4.35%	<0.001
	Scans	83.33%	13.94%	<0.001
	Observations	66.67%	0%	<0.001
	Drug History	88.89%	13.04%	<0.001
	Antibiotics	83.33%	13.04%	<0.001
	Past Medical History	50%	17.39%	0.012
Whether doctors felt this information would be useful at the patient bedside	Yes	97.22%	-	
	No	2.78%	-	
Whether management plans changed when information made available	Yes	75%	21.74%	
	No	13.89%	13.04%	
	N/A	11.11%	65.22%	
Time take to update list	<30 minutes	38.89%	78.26%	0.012
	30-60 minutes	44.44%	17.39%	
	>60 minutes	16.67%	4.35%	
Whether information was adequately conveyed to nursing staff	Yes	13.89%	69.57%	<0.001
	No	86.11%	30.43%	
Whether doctors thought they would be bleeped less	Yes	84.62%	-	
	No	15.38%	-	
Whether doctors were bleeped less	Yes	-	46.15%	
	No	-	53.85%	

There was an increase from 39% to 78% of doctors who spent less than 30 minutes updating a patient list in preparation for ward rounds (p=0.012) (Table 1) while 15.45% more doctors felt the list was more complete by using an electronic system (Figure 3).

By using secure software, such as NerveCentre^TM^, which is available to all members of the MDT, 56% more doctors felt that information from ward rounds was now being adequately communicated to nursing staff (p<0.001). As a result, 46% of doctors said that they were now being bleeped less by staff (Table 1).

The results in Table 2 shows that while consultants and speciality surgical registrar’s agreed that E-Handover improved with regards to information governance, they did not feel that E-Handover made a marked change in the safety and efficiency of the surgical department. In some cases, the previous system was preferred. Comparing that to core surgical trainee’s and FY1/FY2’s, it is clear that the junior members of the team had a different experience and an evident improvement in the safety and efficiency of the surgical department was seen, particularly with respect to the efficiency of ward rounds, patient care and transparency.

**Table 2:** Effects of E-Handover and how it was perceived by doctors of different grades

		Consultant		Specialty Surgical Registrar		Core Surgical Trainee		FY1/FY2	
		Paper List	E-Handover	Paper List	E-Handover	Paper List	E-Handover	Paper List	E-Handover
Is system safe and efficient regarding the following:	Information Governance	11.11%	100.00%	25.00%	50.00%	20.00%	80.00%	20.00%	87.75%
	Completeness of List	11.11%	16.67%	16.67%	0.00%	30.00%	60.00%	20.00%	37.50%
	Efficiency of Ward Round	66.67%	50.00%	50.00%	0.00%	20.00%	80.00%	20.00%	62.50%
	Transparency	22.22%	50.00%	41.67%	25.00%	10.00%	100.00%	20.00%	75.00%
	Patient Care	22.22%	33.33%	50.00%	0.00%	20.00%	80.00%	20.00%	62.50%
	Legibility of Notes	0.00%	50.00%	25.00%	25.00%	20.00%	60.00%	0.00%	37.50%
	None of the Above	22.22%	0.00%	33.33%	50.00%	50.00%	0.00%	60.00%	0.00%

## Discussion

Strengths

Using the IHI model for improvement has helped us evaluate our gradual implementation of surgical E-Handover and act on any problems that arise [10]. We have been able to take suggestions and constructive criticism on board at regular intervals and work toward building a system that allows all members of staff to thrive. The hybrid system in phase 1 was identified to be less clinically secure and the junior medical staff rapidly requested the introduction of phase 2 moving the entire surgical department to E-Handover.

By modelling our handover on guidance from RCS England, we have witnessed the importance of an efficient handover on patient care. Patient care is not limited to physical needs but also involves areas such as patient confidentiality. By moving to an electronic system, we have managed to make great strides in protecting patient data.

With the introduction of iPads, we can now access key clinical information from remote locations and review patients’ blood tests and scans in clinical discussions with their consultants in all areas of the hospital. For surgeons, the time between operative cases is commonly utilised to discuss patient care or changes in clinical plans and the use of mobile devices has enabled effective use of this time.

We have also reduced the need to be in crowded MDT rooms to access computers and look through patient notes. This has helped us abide by COVID-19 social distancing measures and help potentially stop the spread of the virus amongst healthcare professionals.

Having up-to-date patient information during ward rounds is crucial in formulating management plans. It allows the senior clinician to communicate a plan to the junior members of the team, which they can accordingly carry out. By having current clinical reports, this process allows ward work to run smoothly. Clinical care and ordering of tests can be carried out in a timely way, thus improving patient flow. While using the paper list, the majority of doctors would need to return to MDT rooms to gather the latest clinical information. Often, on busy ward rounds, this information is only gathered after the ward round has completed and so this new information will need to be fed back to the senior clinician remotely, thus further disrupting and decreasing the efficiency of ward rounds. Frequently, when this information is made available, management plans tend to change. E-Handover supplied doctors with the up-to-date information needed in real time, reducing disruptions to ward rounds.

Updating the patient hand-over list is usually the responsibility of the junior members of the surgical team and occurs at the end of the day. As doctors now had clinical information on their iPads, the need to meticulously update each blood marker for each patient onto a Microsoft Word^TM^ template was not required, as this information was now available at the patient bedside. By having access to an iPad during ward rounds, the team was able to update the list throughout the day, which allowed junior doctors to abide by the EWTD and leave work soon after their shift ends.

One of the measurements used to assess the safety and efficiency of the surgical handover system was transparency. We wanted to ensure that patient information was updated onto a space that was accessible to all members of the MDT. Previously, we observed that a significant amount of clinical information was being entered into the Microsoft Word^TM^ ward lists, which were only accessible to doctors, as opposed to the integrated paper health care records. Having management plans on software that is accessible to all members of the MDT has improved patient care and transparency. Nursing staff are now more aware of daily plans made by the surgical team and are able to assist in carrying out these plans. Previously, these plans will only have been handwritten in patient notes and these can be difficult to read (Figure 1).

Limitations

Change is always difficult and members of staff will understandably be hesitant to alter from a system they have been used to for 20-30 years. Because certain members are not as confident with using IT software, there were initial difficulties with moving to an electronic system. However, by creating environments where we were able to teach the surgical team how to use the system, these problems started to decrease. As junior doctors rotate through specialities every four months, to ensure that the project can continue to flourish, we have developed a teaching programme outlining how E-Handover works. We were able to teach incoming trainees to PAHT how to use NerveCentre^TM^ and created PDF documents that were disseminated to all staff.

Our second questionnaire received 23 responses, as compared to 37 responses in our first questionnaire. Having a 100% response rate would have allowed us to gain more insight into how the new system was being received by all.

Multiple IT software systems are currently in use at PAHT. When short-staffed, it can be difficult to access all these different systems on an iPad while on ward rounds. We are currently working with the IT department to try and pull information from other software, such as blood reports and scan reports, onto NerveCentre^TM^ to make life easier for the doctor.

During our project, we found other limitations and challenges along the way. These are outlined in Appendix E describing our Plan, Do, Study, Act cycles.

## Conclusions

Surgical E-Handover significantly improves the efficiency of surgical ward rounds and improves information governance. E-Handover also supports doctors to abide by social distancing rules when delivering inpatient care and, as a result, helps attempt to decrease the spread of COVID-19 between healthcare workers.

## Figures and Tables

**Figure 4:**
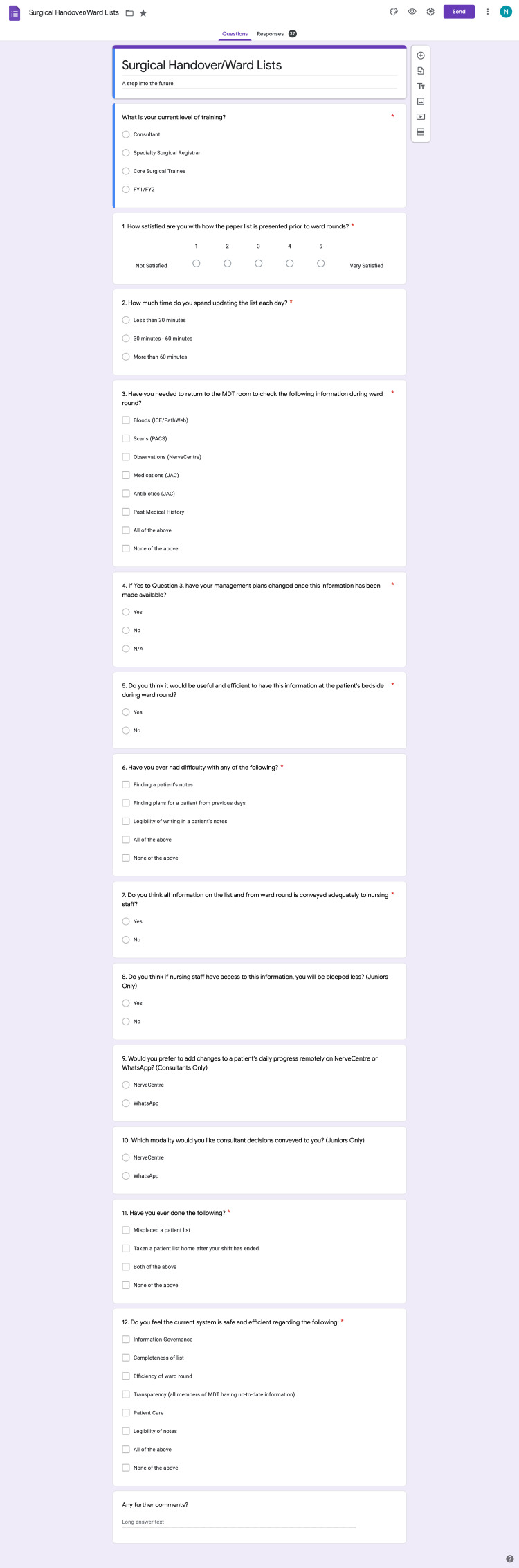
E-Handover Baseline Questionnaire

**Figure 5:**
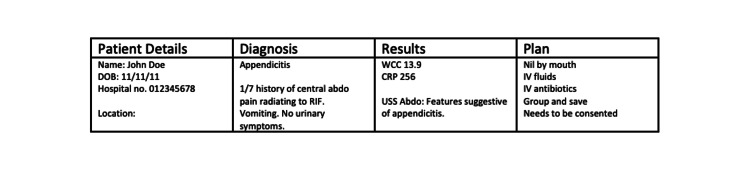
Microsoft Word Handover Template

**Figure 6:**
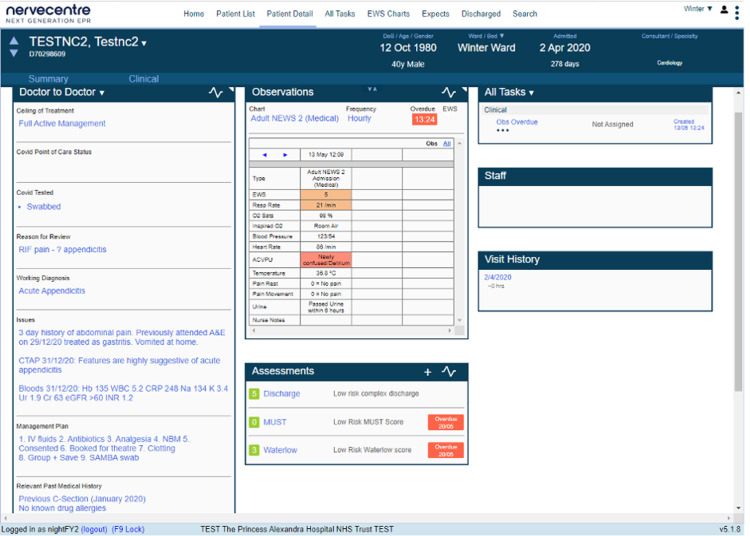
NerveCentre Handover Template

**Figure 7:**
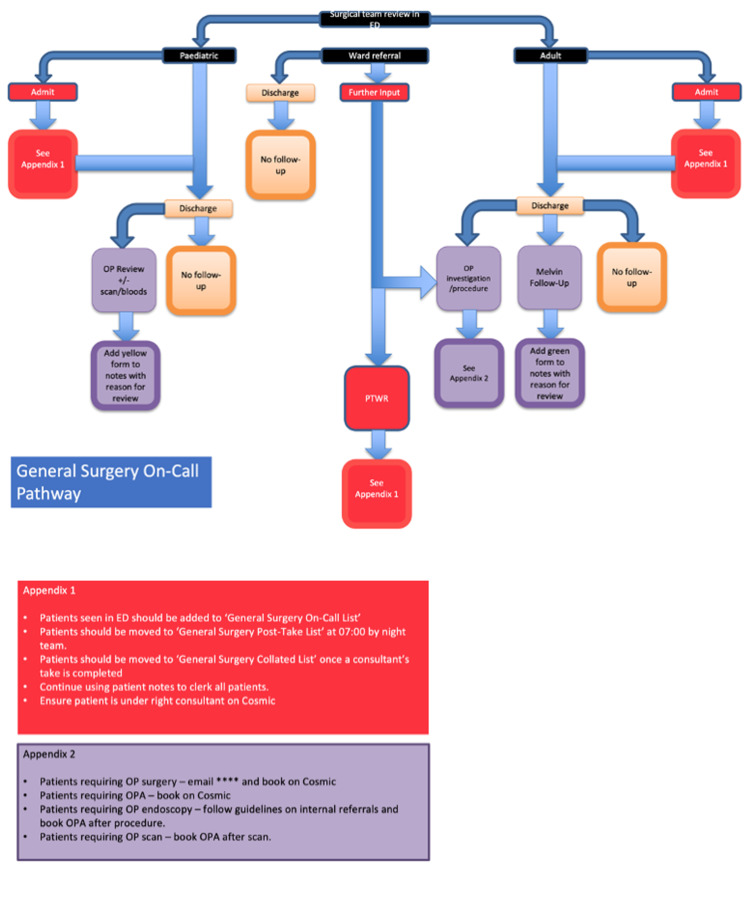
General Surgery E-Handover Flow Chart

## Appendices

Appendix A 

Appendix B 

Appendix C 

Appendix D

Strategy

We used the Plan, Do, Study, Act (PDSA) cycle in The Model of Improvement for our Quality Improvement (QI) project. Given the nature of our project, we felt it was an excellent framework to use so that we could evaluate the ongoing minor changes we made before permanently moving to a new system. By doing this, we felt we would be in the best position to minimise disruption for both staff and patients and to not compromise patient care.

PDSA Cycle 1:* *We were given five iPad’s by the IT department, who modified them to include NerveCentre^TM^ and enable us to check blood test reports, imaging, observations and patient medications via our electronic prescribing and medicines administration software. The iPads also allowed us to prescribe medications and request any required scan.

Five iPad’s were distributed to the five members of the on-call team, as they were being trialled for on-call purposes alone and not for the ward doctors.

We worked with the IT team to create two new patient lists on NerveCentre^TM^, which were the ‘General Surgery On-Call List’ and ‘General Surgery Post-Take List’. Once a patient was seen in the Accident and Emergency Department (A+E), they were added to the ‘General Surgery On-Call List’ if further surgical input was required. This would occur over a 24-hour period from 08:00 to 08:00 the following day. Prior to morning handover, the patients were moved from the ‘General Surgery On-Call List’ to the ‘General Surgery Post-Take List’, and the on-call team would handover using their iPads while maintaining social distancing. At 17:00, patients would then be added to the ward paper lists on Microsoft Word^TM^ and removed from the NerveCentre^TM^ ‘General Surgery Post-Take List’. 

PDSA Cycle 2: After success using the iPads for the surgical handovers, we soon realised that it was impractical and more time-consuming to transfer patients from an electronic on-call system to the ward’s paper system. We expanded the project to include all surgical ward work and requested five new iPads and a laptop to aid the ward doctors with their ward rounds and subsequent jobs. We generated a new patient list on NerveCentre^TM^: ‘General Surgery Collated List’. Instead of moving patients from the Post-Take List to a ward's paper list, they were moved to the ‘General Surgery Collated List’. This process was put into a flowchart and taught to the department at the weekly surgical breakfast meeting (see Appendix E).

PDSA Cycle 3: We encountered difficulties during our QI project, the first being a technical issue due to multiple software programmes being used at PAHT. Patients seen in A+E requiring admission under the surgical team would momentarily be ‘discharged’ from A+E and the hospital before being re-admitted back onto a surgical bed. This was being classed as two separate admissions to the hospital. As a result, all clinical information that had been written onto IT software while the patient was in A+E would be removed and would need to be re-written when they had moved onto the ward. The team found this particularly challenging, especially during busy on-call shifts. We discussed this with the IT department and a solution was found to class the given example as one admission to hospital but with 2 separate episodes. This allowed a patient’s clinical information and medications to stay on the system as long as they were in the hospital. 

PDSA Cycle 4: During the height of the pandemic, there were very few surgical ward patients and so a designated consultant or senior registrar would see all admitted surgical patients. With COVID-19 cases decreasing and surgical referrals and admissions rising, PAHT moved back to a larger surgical team with an on-call team, post-take team and ward team. Junior doctors who had been re-deployed moved back to the surgical department and consultants restarted their usual on-call rota. As a result, the consultants would need to know which patients had come in during their on-call so they could take responsibility for their management. Often, when a patient is admitted under the surgical team, they are admitted under the incorrect consultant and so the patient would not appear on the appropriate NerveCentre^TM^ list, leading to an interruption in ward rounds. Again, we liaised with the IT department regarding the issue, and we were shown how to manually change the admitting consultant, which would allow the patient to be under the appropriate consultant list on NerveCentre^TM^. This was taught to all members of the surgical department, and a step-by-step PDF document was shared with staff.

Appendix E
